# Adiponectin Inhibits the Production of TNF-α, IL-6 and Chemokines by Human Lung Macrophages

**DOI:** 10.3389/fphar.2021.718929

**Published:** 2021-08-23

**Authors:** Hélène Salvator, Stanislas Grassin-Delyle, Marion Brollo, Louis-Jean Couderc, Charlotte Abrial, Tatiana Victoni, Emmanuel Naline, Philippe Devillier

**Affiliations:** ^1^ Laboratory of Research in respiratory Pharmacology- Virologie et Immunologie Moleculaire (VIM)- UMR 0892 Université Paris-Saclay, Suresnes, France; ^2^ Faculté des Sciences de la Santé Simone Veil, UVSQ Paris‐Saclay University, Montigny‐le‐Bretonneux, , France; ^3^ Department of Respiratory Diseases, Foch Hospital, Suresnes, France; ^4^ Mass Spectrometry Platform and INSERM UMR1173, Montigny-le-Bretonneux, France; ^5^ University of Lyon, VetAgro Sup, APCSe, Marcy l’Étoile, France

**Keywords:** adiponectin, obesity, human lung macrophage, cytokine, adiporon, LPS, Poly (I:C)

## Abstract

**Background:** Obesity is associated with an elevated risk of severe respiratory infections and inflammatory lung diseases. The objectives were to investigate 1) the production of adiponectin by human lung explants, 2) the expression of the adiponectin receptors AdipoR1 and AdipoR2 by human lung macrophages (LMs), and 3) the impact of recombinant human adiponectin and a small-molecule APN receptor agonist (AdipoRon) on LMs activation.

**Material and methods:** Human parenchyma explants and LMs were isolated from patients operated for carcinoma. The LMs were cultured with recombinant adiponectin or AdipoRon and stimulated with lipopolysaccharide (10 ng ml^−1^), poly (I:C) (10 µg ml^−1^) or interleukin (IL)-4 (10 ng ml^−1^) for 24 h. Cytokines or adiponectin, released by explants or LMs, were measured using ELISAs. The mRNA levels of AdipoR1 and AdipoR2 were determined using real-time quantitative PCR. AdipoRs expression was also assessed with confocal microscopy.

**Results:** Adiponectin was released by lung explants at a level negatively correlated with the donor’s body mass index. AdipoR1 and AdipoR2 were both expressed in LMs. Adiponectin (3–30 µg ml^−1^) and AdipoRon (25–50 μM) markedly inhibited the LPS- and poly (I:C)-induced release of Tumor Necrosis Factor-α, IL-6 and chemokines (CCL3, CCL4, CCL5, CXCL1, CXCL8, CXCL10) and the IL-4-induced release of chemokines (CCL13, CCL17, CCL22) in a concentration-dependent manner. Recombinant adiponectin produced in mammalian cells (lacking low molecular weight isoforms) had no effects on LMs.

**Conclusion and implications:** The low-molecular-weight isoforms of adiponectin and AdipoRon have an anti-inflammatory activity in the lung environment. Targeting adiponectin receptors may constitute a new means of controlling airways inflammation.

## Introduction

In a worldwide analysis in 2016, it was estimated that about 671 million adults and 124 million children over the age of four were obese, and that a further 1.3 billion adults and 213 million children over the age of four were overweight (NCD Risk Factor Collaboration (NCD-RisC), 2017).

Overweight and obesity are associated with an increased risk of noncommunicable diseases such as diabetes mellitus and cardiovascular disease (GBD 2015 Obesity Collaborators et al., 2017). Obesity also constitutes a risk factor for respiratory infections such as pneumonia, severe influenza and severe coronavirus disease 2019 (Baik et al., 2000; Morgan et al., 2010; Salvator et al., 2011; Van Kerkhove et al., 2011; Kass and Priya, 2020; Lighter et al., 2020). Epidemiological studies have demonstrated that 1) asthma is more likely to occur in obese individuals, and 2) obese people with asthma experience more severe symptoms, worse quality of life, and increased healthcare use (Sutherland 2014).

Fat tissue acts as an endocrine organ by releasing various bioactive substances referred to collectively as adipokines (Ouchi et al., 2011; Leal et al., 2013). Adiponectin (APN, the most abundant adipokine) is known to deactivate proinflammatory pathways (Ouchi et al., 2011; Ohashi et al., 2014). In lean individuals, APN is present in the circulation at high concentrations (∼µg/ml) in various oligomeric states. However, circulating levels of APN are lower in obese individuals, and this fall is thought to contribute to obesity-related inflammatory diseases (Arita et al., 1999; Ouchi et al., 2011).

A growing body of evidence suggests that APN modulates macrophage function. Macrophages can be committed to a continuum of functional phenotypes. The two extremes of the spectrum are the phenotypes often referred as either M1 or M2. Stimulation of toll-like receptor 4 (TLR4) by lipopolysaccharide (LPS) or of TLR3 by poly (I:C) to mimic a bacterial or a viral infection respectively, leads to a classical macrophage activation state (M1) with the production of cytokines (such as tumor necrosis factor-α (TNF-α) and IL-6) and a particular subset of CC and CXC chemokines. The Th2 cytokines (IL-4, IL-13) generate an alternative macrophage activation state (M2) in which a different subset of chemokines is produced (Murray et al., 2014; Abrial et al., 2015; Shapouri-Moghaddam et al., 2018).

Adiponectin’s effect on cytokine production by monocytes-macrophages has been examined in nonstimulated or LPS-stimulated preparations. In LPS and interferon-γ (M1)-activated murine bone-marrow-derived macrophages, APN exerted a proinflammatory effect by substantially elevating the expression of TNF-α, IL-6, and IL-12 levels (van Stijn et al., 2015). In human monocyte-derived macrophages (MDMs), the results of transcriptional profiling experiments indicated that APN promotes a pro-inflammatory phenotype which resembles M1 more than it does M2 (Cheng et al., 2012). In contrast to the latter study on unstimulated MDM, experiments in porcine or human MDMs have shown that APN abrogates the LPS-stimulated expression or production of IL-6, TNF-α, CCL2 and CXCL10 (Yokota et al., 2000; Wulster-Radcliffe et al., 2004; Okamoto et al., 2008; Ohashi et al., 2010). At present, however, little is known about APN’s actions on lung macrophages. Mouse alveolar macrophages express the two main specific APN receptors (AdipoR1 and AdipoR2) and inhibits the LPS-mediated expression or release of TNF-α and CCL2 (Summer et al., 2008; Ohashi et al., 2010). Mouse macrophages and human MDMs are both surrogate cell models that do not adequately recapitulate the biology of human primary lung macrophages (LMs). Adiponectin’s known role in obesity-related pulmonary inflammation and diametrically opposing effects of APN on different macrophage preparations prompted us to investigate 1) APN production by human lung explants, 2) the expression of the two AdipoRs on LMs, and 3) the impact of recombinant APNs and a small-molecule APN receptor agonist (AdipoRon (Okada-Iwabu et al., 2013)) on the regulation of LM activation by LPS, poly (I:C) or IL-4.

## Material and Methods

### Materials

Human recombinant APNs (expressed either in *Escherichia coli* (*E.coli*) or in human embryonic kidney (HEK) 293 cells) were purchased from Biovendor (Karasek, Czech Republic). AdipoRon hydrochloride and human recombinant IL-4 (produced in *E.coli*) were acquired from TOCRIS/R&D Systems (Lille, France) and solubilized respectively in DMSO and Roswell Park Memorial Institute 1,640 medium (RPMI). Antibiotics, DMSO, l-glutamine, trypan blue dye, heat-inactivated fetal calf serum and LPS (from *E. coli* serotype 0111:B4) were purchased from Sigma (St. Louis, MO, United States). High-molecular-weight poly (I:C) was obtained from InvivoGen (Toulouse, France). Bovine serum albumin and RPMI medium were from Eurobio Biotechnology (Les Ulis, France).

### Preparations of Human Lung Explants and Macrophages

**FIGURE 1:**
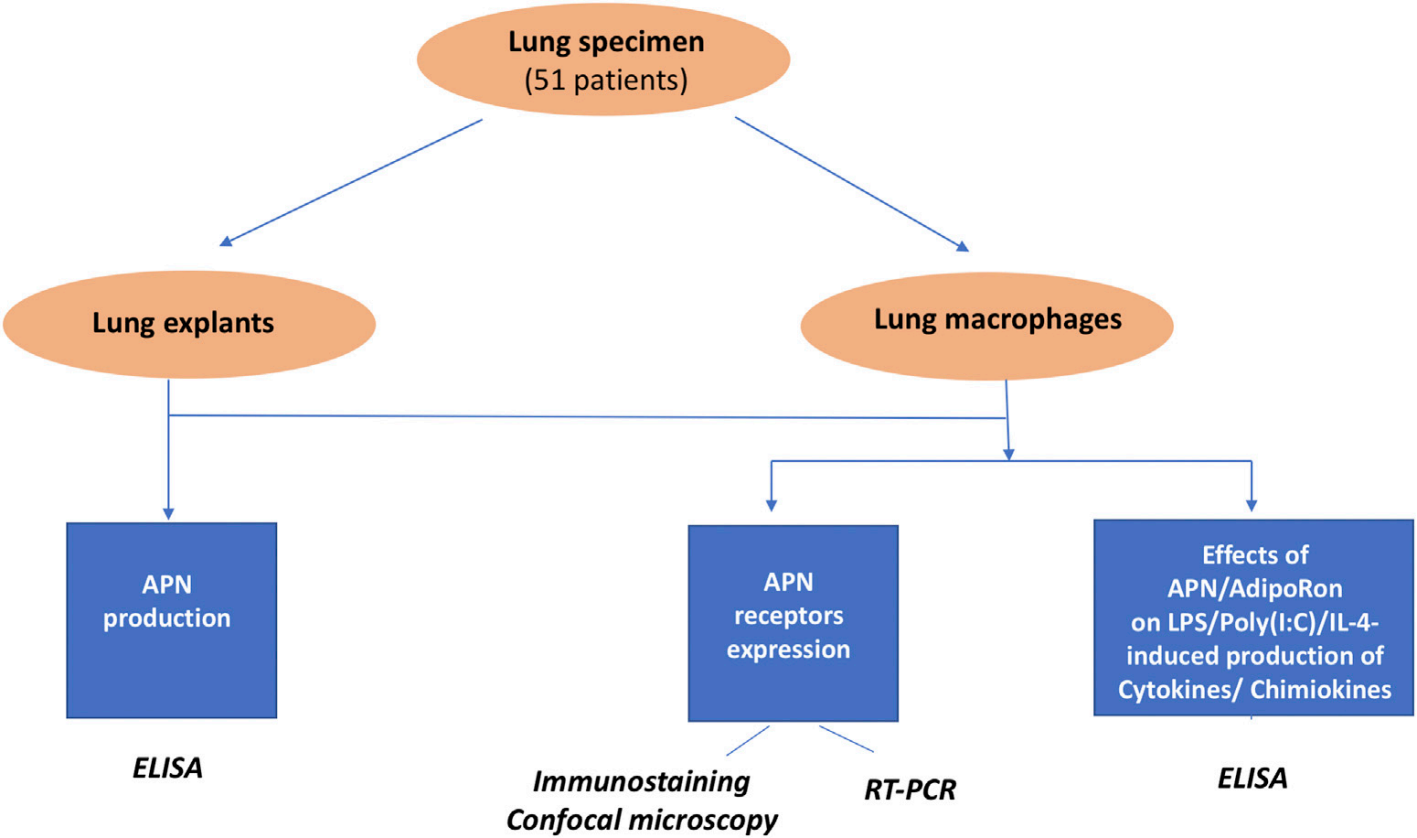
Experimental design.

The design of the study is summarized in Figure 1.

Experiments on human tissue were approved by the regional investigational review board (*Comit*é *de Protection des Personnes* Îl*e de France VIII*, Boulogne-Billancourt, France). Lung tissue samples were obtained from 51 patients (median age: 63 years [range 49–83]; 32 males and 19 females; current smoker/ex-smoker: 49; never smoker: 2; mean ± standard deviation (SD) pack-years: 40 ± 22; BMI: 25 ± 5; FEV1: 87 ± 16%; FEV_1_/FVC ratio: 0.78 ± 0.14) undergoing surgical resection for lung carcinoma and who had not received prior chemotherapy. The lung explants and LMs were isolated from macroscopically normal lung parenchyma obtained from sites distant from the tumor, dissected free of pleura, visible airways and blood vessels as previously described (Buenestado et al., 2010; 2012; 2013).

#### Human Lung Explants

Human lung parenchyma was prepared after several washes of finely chopped into 3–5 mm^3^ parenchymal fragments in complete culture medium (RPMI 1640, supplemented with 2 mM l-glutamine, 100 µg ml^−1^ streptomycin, and 100 U.ml^−1^ penicillin), in order to prevent contamination by blood. The explants were maintained overnight at 4°C. The fragments (total weight ∼50–100 mg each) were distributed into six-well plates (five fragments and 5 ml of complete medium per well). After 2, 4 and 24 h of incubation in RPMI at 37°C (in a 5% CO_2_ humidified atmosphere), culture supernatants from the explants were collected and stored at -80°C for subsequent APN assays.

#### Human Lung Macrophages

Lung macrophages were isolated by adherence, as previously described (Abrial et al., 2015). Briefly, the fluid collected from several washings of minced peripheral lung tissues was centrifuged (2000 rpm for 10 min). The cell pellet was resuspended in RPMI supplemented with 10% heat-inactivated fetal calf serum, 2 mM l-glutamine and antibiotics. Resuspended viable cells (106 per ml) were then aliquoted into either a 12-well plate for transcriptional assays or a 24-well plate for cytokine assays. Following incubation for at least 1.5 h at 37°C in a 5% CO2 humidified atmosphere, non-adherent cells were removed by gentle washing. The remaining cells were maintained at 37°C and 5% CO2 overnight. It has been shown that the adherence step does not significantly influence the overall transcriptional changes in alveolar macrophages, relative to flow-cytometry-based isolation (Shaykhiev et al., 2009). The adherent cells (211 ± 54 × 10^3^ cells per well, for a 24-well plate) were >95% pure macrophages, as determined by May-Grünwald-Giemsa staining and CD68 immunocytochemistry. Cell viability exceeded 90%, as assessed by trypan blue dye exclusion. Culture plates with adherent macrophages were washed with warm medium. One mL of fresh medium supplemented with 1% fetal calf serum was added per well, and culture plates were incubated overnight at 37°C in a 5% CO_2_ humidified atmosphere.

### Treatment of Lung Macrophages and Explants

On the day after isolation, macrophages or explants were washed twice, and 1 ml of RPMI was added per well. Classically activated macrophages were obtained by exposure for 24 h to LPS (10 ng mL^−1^) or poly (I:C) (10 µg ml^−1^), and alternatively activated macrophages were produced by exposure to IL-4 (10 ng ml^−1^). The LPS concentration (10 ng ml^−1^) was selected as suboptimal on the basis of time-response and concentration-response curves from preliminary experiments (Buenestado et al., 2013). The poly (I:C) concentration (10 µg ml^−1^) was chosen on the basis of preliminary experiments in which it elicited much the same level of cytokine production as LPS 10 ng ml^−1^. IL-4 concentration was chosen on the basis of 1) literature data from human macrophage models (Pechkovsky et al., 2010; Staples et al., 2012) and 2) preliminary experiments in our laboratory (Abrial et al., 2015).

Recombinant APN (3–10–30 µg ml^−1^) or AdipoRon (5–10–25–50 µM) was added to the culture medium 1 hour before exposure to LPS, poly (I:C) or IL-4. The APN concentrations were originally chosen to reflect human plasma concentrations (3–30 μg ml^−1^) (Arita et al., 1999; Lindberg et al., 2013; Lindberg et al., 2017). In control experiments, the vehicle used for AdipoRon (0.1% DMSO) did not alter cytokine production. Following 24 h of incubation, supernatants were collected and stored at -80°C for subsequent analysis.

### Cytokine and Adiponectin Assays

The supernatants’ cytokine concentrations were measured with an ELISA (R&D Systems), according to the manufacturer’s instructions. The limits of detection was 4 pg ml^−1^ for CCL3, 8 pg.ml-^1^ for CCL17, CCL13 and CCL22, TNF-α, CCL2, CCL5 and CCL4, 9.4 pg ml^−1^ for IL-6 and 32 pg ml^−1^ for CXCL1, CXCL8 and CXCL10.

The supernatants were diluted with RPMI as appropriate, and the optical density was determined at 450 nm using a microplate reader (MRX II, Dynex Technologies, Saint-Cloud, France). Cytokine concentrations are expressed in ng.10^−6^ LMs.

The APN levels were measured with an ELISA (R&D Systems; detection range: 62.5 – 4,000 pg ml^−1^). Cell viability was assessed by measuring LDH release with the CytoTox96^®^ Non-Radioactive Cytotoxicity Assay (Promega^®^, Madison, United States) in the LMs supernatants (Supplemental material).

### Quantitative Reverse Transcriptase Polymerase Chain Reaction

Lung macrophages were stimulated (or not) for 24 h with LPS, poly (I:C) or IL-4. Total RNA was prepared using TRIzol^®^ reagent (Life Technologies, Saint Aubin, France), according to the manufacturer’s instructions. The RNA’s intactness was determined by running an aliquot of each sample on an ExperionTM automated electrophoresis station (Bio-Rad, Marnes-la-Coquette, France). Next, 1 µg of total RNA was reverse-transcribed using SuperScript^®^ III First-strand SuperMix kit (Life Technologies) prior to analysis with specific TaqMan^®^ arrays.

Specific TaqMan^®^ arrays based on predesigned reagents (*AdipoR1*: Hs01114951_m1; *AdipoR2*: Hs00226105_m1, Thermo Fisher Scientific, MA, United States) were used to analyze AdipoR1 and AdipoR2 transcripts. Reverse transcriptase-quantitative polymerase chain reaction was performed using Gene Expression Master Mix (Thermo Fisher Scientific) with 20 ng of cDNA in a StepOnePlus thermocycler (Thermo Fisher Scientific). The thermal cycling conditions were as follows: initial denaturation at 95°C for 10 min, followed by 40 cycles of 95°C for 15 s and 60°C for 1 min. The housekeeping gene coding for hypoxanthine phosphoribosyltransferase (*HPRT1*: Hs99999909_m1) was used for signal normalization.

### Laser Confocal Immunofluorescence Microscopy

The LMs were fixed in methanol 80% on labteck glass chamber slides. After incubation with 1% bovine serum albumin in phosphate buffered saline solution for 30 min, immunostaining was performed using primary antibodies targeting either AdipoR1 (monoclonal rabbit Ig, dilution 1/100; ENZO, Lausanne, Switzerland) or AdipoR2 (monoclonal rabbit Ig, dilution 1/30; Abcepta, CA, United States). After 1 h of incubation, a secondary antibody (donkey monoclonal anti rabbit Ig) coupled with fluorescent probe Alexa Fluor 488 (green) was added for 1 h before washing. Images were acquired using a SP5 Leica confocal microscope (Leica, Nanterre, France) with a 63X objective. A spectral imaging acquisition method was used in order to overcome the challenge of spectral overlap with macrophages autofluorescence.

### Statistical Analysis

Data are expressed as the mean ± SEM per 10^6^ macrophages or per 100 mg lung explants obtained from n patients. The APN concentrations in the supernatants of the lung explants were compared using either paired Student’s t test or an ANOVA followed by Tukey’s post-test for multiple comparisons for the time-course experiment. The relationship with BMI was estimated by calculating Spearman’s correlation coefficient. The statistical significance of the effects of LPS, poly (I:C) or IL-4 on the cytokine production and on the expression of both AdipoR1 and AdipoR2 transcripts was assessed using either paired or unpaired Student’s t test as appropriate. The effects of various concentrations of APN and AdipoRon on cytokine levels released by LMs were compared on log-transformed data using a one-way repeated measures ANOVA followed by Dunnett’s post-test for multiple comparisons. Statitiscal analyzes were performed using GraphPad Prism^®^ software (version 7, GraphPad Software Inc., San Diego, CA, United States). The threshold for statistical significance was set to *p* < 0.05.

## Results

### Production of Adiponectin by Human Lung Explants

The APN concentration in the supernatant above nonstimulated explants (n = 30 patients) after 24 h was 185.0 ± 127.3 ng ml^−1^ per 100 mg of tissue. After adjustment for the volumes of the culture medium (3 ml) and the explants (100 mg ≈ 0.1 ml), this corresponded to a concentration of APN about 6.1 µg g^−1^ of lung tissue (i.e. close to the blood concentration). Neither LPS, poly (I:C) nor IL-4 altered APN production.

**FIGURE 2:**
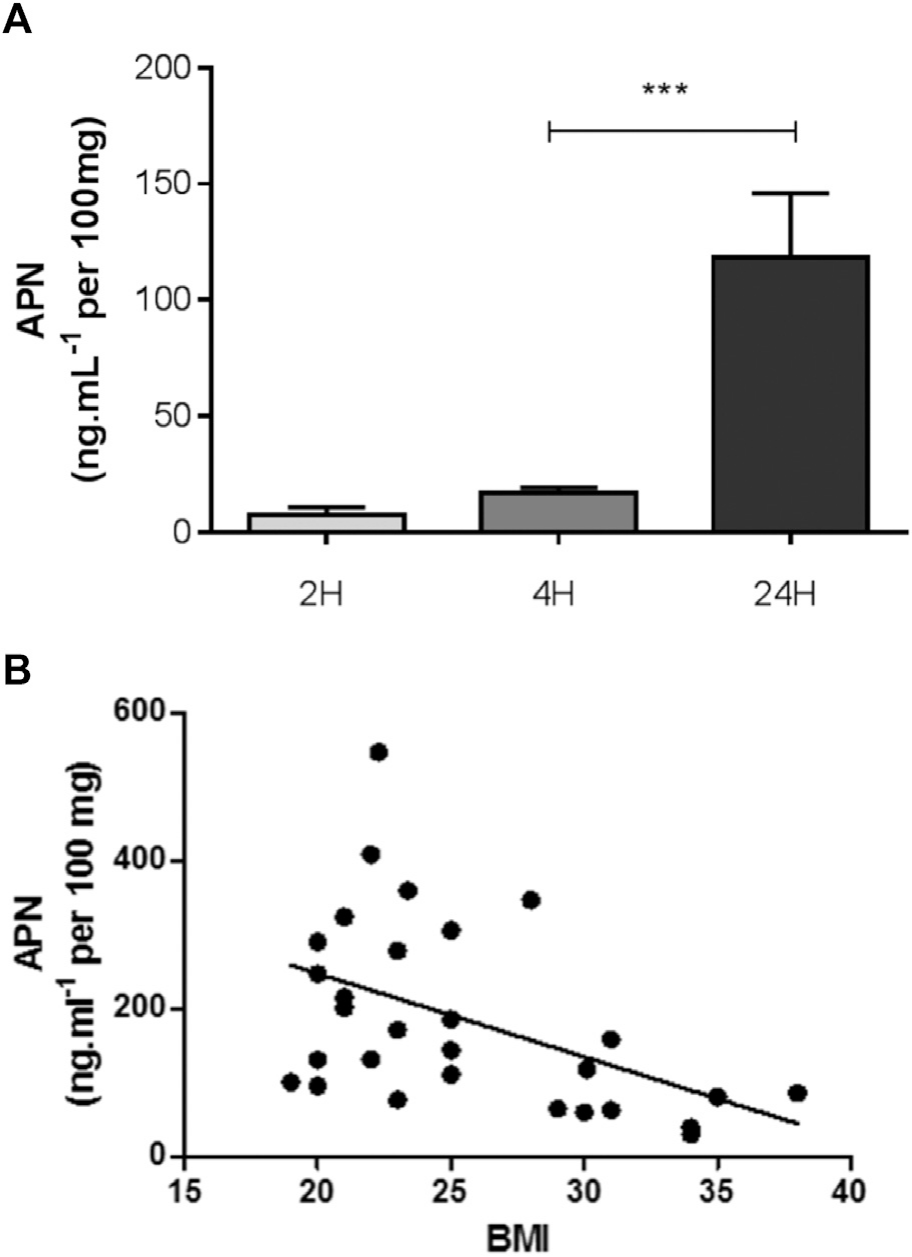
APN release from lung explants. **(A)** Time-course of APN release from nonstimulated human lung explants cultured for 2, 4 and 24 h. Data are reported as the mean ± SEM of 5–11 independent experiments (***: *p* < 0.001). **(B)** Relationship between the APN production from unstimulated human lung explants after 24 h incubation assessed as the concentration in the supernantants and the donor’s BMI (n = 30). Spearman’s correlation coefficient r = -0.48; p = 0.008.

Time-course measurements of the APN concentration in explant supernatants highlighted an increase over time, and suggested that APN was released by the tissue (Figure 2A). The APN concentrations were negatively correlated with the explant donor’s BMI (Figure 2B). In contrast, APN was not detected in LM supernatants - suggesting that these macrophages were not involved in the release of APN by the explants.

### Expression of Adiponectin Receptors on Human Lung Macrophages

**FIGURE 3:**
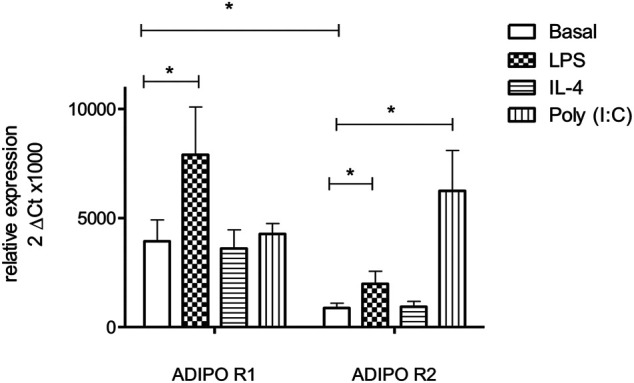
Expression of AdipoR1 and AdipoR2 transcripts by human LMs. Human LMs were cultured for 24 h in absence or presence of LPS (10 ng ml^−1^), poly (I:C) (10 µg ml^−1^) or IL-4 (10 ng ml^−1^). AdipoR1 and AdipoR2 transcript levels were determined by RT-qPCR and normalized against those of a housekeeping gene (*HPRT1*). Data correspond to the mean ± SEM of 6 independent experiments (*: *p* < 0.05).

**FIGURE 4:**
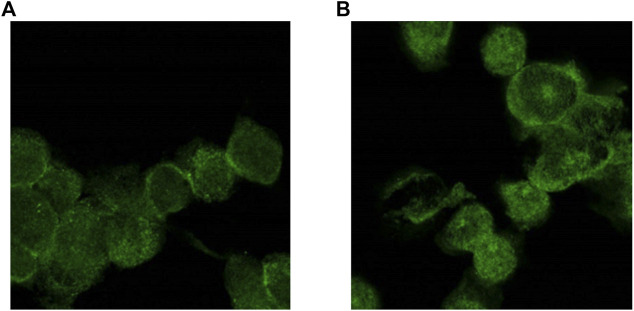
AdipoR1 and AdipoR2 are expressed on human LMs. AdipoR1 **(A)** and AdipoR2 **(B)** staining appears in green (AF488 fluorochrome). Immunostaining was evaluated using a SP5 Leica confocal microscopy (X63) and a spectral imaging acquisition.

An RT-PCR analysis demonstrated that LMs expressed both AdipoR1 and AdipoR2 at the mRNA level. Unstimulated LMs expressed AdipoR1 more strongly than AdipoR2. The expression of both AdipoR1 and AdipoR2 transcripts was enhanced after exposure to LPS for 24 h, whereas exposure to poly (I:C) only enhanced the expression of AdipoR2. In contrast, IL-4 did not alter the transcription of either APN receptor (Figure 3). The expression of the two types of AdipoRs was also detected by immunostaining and confocal microscopy (Figure 4).

### Adiponectin and AdipoRon Abrogate the LPS- and Poly(I:C)-Induced Production of Cytokines by Lung Macrophages

#### LPS- and poly(I:C)-Induced Cytokine Production by Lung Macrophages

Incubation with LPS or poly (I:C) during 24 h was associated with markedly greater production of various M1 cytokines (Table 1). Exposure to LPS was associated with greater production of TNF-α, IL-6, CXCL1, and CXCL8, whereas exposure to poly (I:C) was associated with greater production of CCL5 and CXCL10. Conversely, LPS and poly (I:C) did not alter (or only weakly altered) the production of the M2 chemokines CCL13, CCL17, and CCL22 (data not shown) (Abrial et al., 2015).

**TABLE 1:** Amounts of cytokines in the supernatants of human LMs treated with LPS or poly (I:C).

Cytokine	Baseline	LPS 10 ng.ml-1	poly (I:C) 10 µg.ml-1	LPS vs poly (I:C)
**CCL3**	**1.9 ± 0.3**	191.8 ± 34.5*** [101]	81.9 ± 14.5*** [43]	**ns**
**CCL4**	**4.1 ± 2.1**	197.5 ± 80.4*** [48]	178.1 ± 25.5*** [43]	**ns**
**CCL5**	**0.07 ± 0.05**	1.1 ± 0.4*** [15]	3.5 ± 0.4*** [49]	*
**TNF-alpha**	**0.08 ± 0.1**	33.2 ± 5.7 *** [415]	8.4 ± 1.8** [105]	*
**IL-6**	**0.35 ± 0.18**	81.3 ± 17.2*** [232]	2.7 ± 0.9* [8]	**
**CXCL1**	**0.85 ± 0.4**	155.6 ± 22.9*** [183]	7.1 ± 2.6* [8]	***
**CXCL8**	**23.0 ± 2.9**	989.6 ± 152.8*** [43]	72.7 ± 20.5** [3]	***
**CXCL10**	**0.03 ± 0.007**	2.33 ± 0.7** [78]	14.6 ± 2.3*** [487]	**

Results are expressed as ng/10^6^ LMs and reported as the mean ± SEM of 5–16 independent experiments. The increase in the production of the cytokines induced by LPS or poly(I:C) is expressed as fold-change relative to baseline and given in brackets. Asterisks indicate significant differences relative to the baseline condition or a significant difference between LPS and poly(I:C) (^*^: *p* < 0.05; ^**^: *p* < 0.01; ^***^: *p* < 0.001).

#### Effect of Adiponectin on the LPS- and Poly(I:C)-Induced Production of Cytokines by Lung Macrophages

**FIGURE 5:**
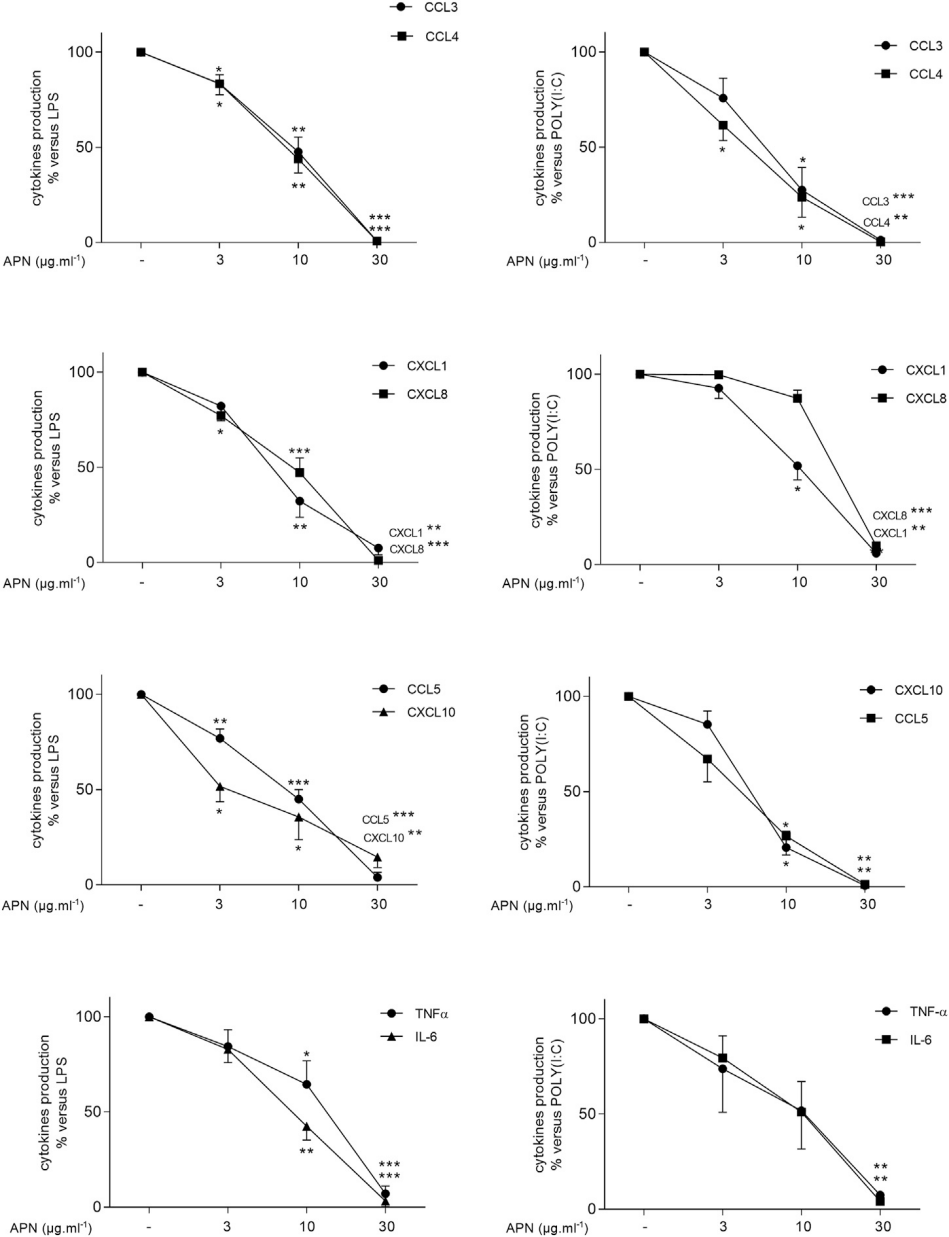
Inhibitory effects of APN on the LPS- or poly(I:C)-induced cytokine release by human LMs. Macrophages were incubated with LPS (10 ng ml^−1^, left column) or poly (I:C) (10 µg ml^−1^, right column) in the absence or presence of APN (3, 10 or 30 µg ml^−1^) for 24 h. Data are expressed as a percentage with respect to LPS or poly (I:C)-induced production. Results are shown as the mean ± SEM of 5–16 different experiments. Asterisks indicate significant effects of APN with respect to LPS or poly (I:C) alone (*:*p* < 0.05; **: *p* < 0.01; ***: *p* < 0.001).

On unstimulated LMs, APN did not alter the production of cytokines (Supplementary Table S1). Adiponectin inhibited both LPS- and poly (I:C)-stimulated cytokine production in a concentration-dependent manner (Figure 5 and Supplementary Table S2). A submaximal concentration (10 µg ml^−1^) of APN had similar effects on the LPS- and poly (I:C)-induced production of cytokines, with the exception of CXCL8. Although APN (10 µg ml^−1^) markedly inhibited the LPS-induced CXCL8 production, it did not change significantly the poly (I:C)-induced CXCL8 production (Figure 5 and Supplementary Table S2).

At a concentration of 30 µg ml^−1^, APN almost completely inhibited the cytokine production (Supplementary Table S2) and was associated with a 70% relative reduction in the LPS-induced production of IL-10 (Supplementary Material).

#### Effects of Various Recombinant Adiponectins on the LPS-Induced Production of Cytokines by Lung Macrophages

In addition to human recombinant APN produced in *E. coli*, we tested recombinant APN produced in HEK293 cells (Biovendor). The main difference between the two was the presence of high-molecular-weight (HMW) isoforms (more than hexameric) in the HEK293 APN. The APN expressed in *E. coli* lacks the characteristic post-translational modifications of eukaryotic APN and particularly fails to form HMW multimers (Tsao et al., 2002). When tested on unstimulated LMs (Supplementary Table S1), APN from HEK293 cells was devoid of significant effects on the production of TNF-α, CXCL8, CCL3, CCL4, or IL-6 at concentrations up to 30 µg ml^−1^ (n = 5–6) but significantly inhibited the LPS-induced production of TNF-α and CCL4 at 30 µg ml^−1^ (Supplementary Table S3).

A mutant APN (C39A: alanine substituted for cysteine at position 39) expressed in HEK293 cells (Biovendor) forms trimers but not hexamers or HMW isoforms (Haugen et al., 2007). At 30 µg ml^−1^, the mutant APN did not alter the production of cytokines by unstimulated or LPS-stimulated LM (n = 5). This latter result agrees with previous reports on human blood monocytes and U937 monocytic cells (Haugen et al., 2007; Song and Chan, 2009).

We also compared the activity of several batches of APN produced in *E. coli* (Biovendor). One batch clearly differed in its low-molecular-weight (LMW) isoform content (Supplementary Figure S1) and was devoid of inhibitory activity on LPS-induced cytokine production. Only the batches containing the LMW isoforms (used in the present study) inhibited the LPS-induced production of cytokines. As a whole, these findings suggest that APN’s anti-inflammatory activity can be ascribed to its LMW isoforms - probably the hexamers.

#### Effects of AdipoRon on the LPS and Poly(I:C)-Induced Production of Cytokines by Lung Macrophages

**FIGURE 6:**
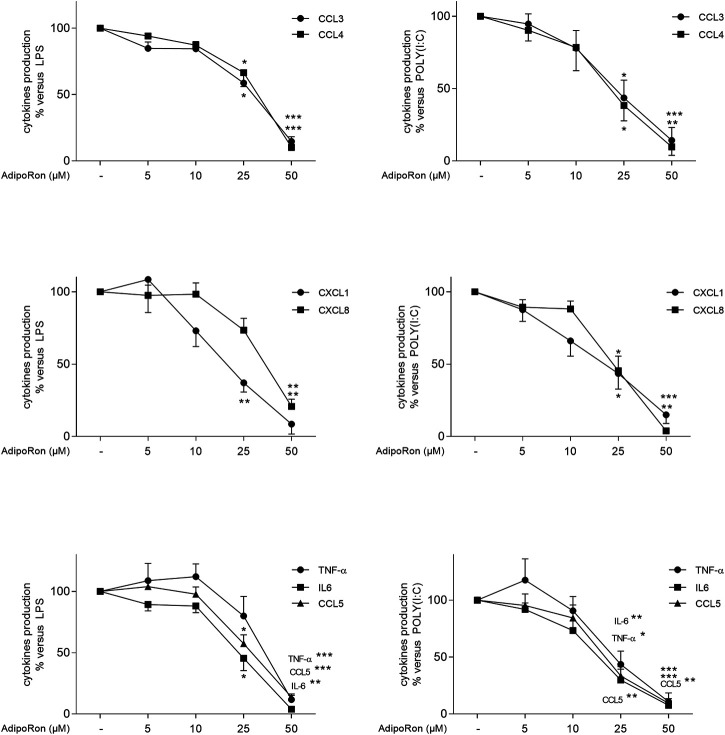
Inhibitory effects of AdipoRon on the LPS- or poly(I:C)-induced cytokine release by human LMs. Macrophages were incubated with LPS (10 ng ml^−1^, left column) or poly (I:C) (10 µg ml^−1^, right column) in the absence or presence of AdipoRon (5, 10, 25, and 50 µM). Data are expressed as a percentage with respect to LPS or poly (I:C)-induced production. Results are shown as the mean ± SEM of 5–9 different experiments. Asterisks indicate significant effects of AdipoRon with respect to LPS or poly (I:C) alone (*:*p* < 0.05; **: *p* < 0.01; ***: *p* < 0.001).

On unstimulated LMs, AdipoRon inhibited the basal production of TNF-α (*p* < 0.05), IL-6 (*p* < 0.05), CXCL1 and CXCL8 (Supplementary Table S1), AdipoRon also inhibited LPS- and poly (I:C)-induced cytokine production in a concentration-dependent manner (Figure 6 and Supplementary Table S4). AdipoRon at concentrations of 5–50 µM activate the APN receptors in myotubes to the same extent as APN does (Okada-Iwabu et al., 2013). As observed with APN, AdipoRon was less effective in inhibiting poly (I:C)-induced CXCL8 production. The inhibitory effects of AdipoRon (50 µM) did not differ significantly from those of APN (30 µg ml^−1^).

### Effects of Adiponectin and AdipoRon on the IL-4-Induced Release of Cytokines by Human Lung Macrophages

**FIGURE 7:**
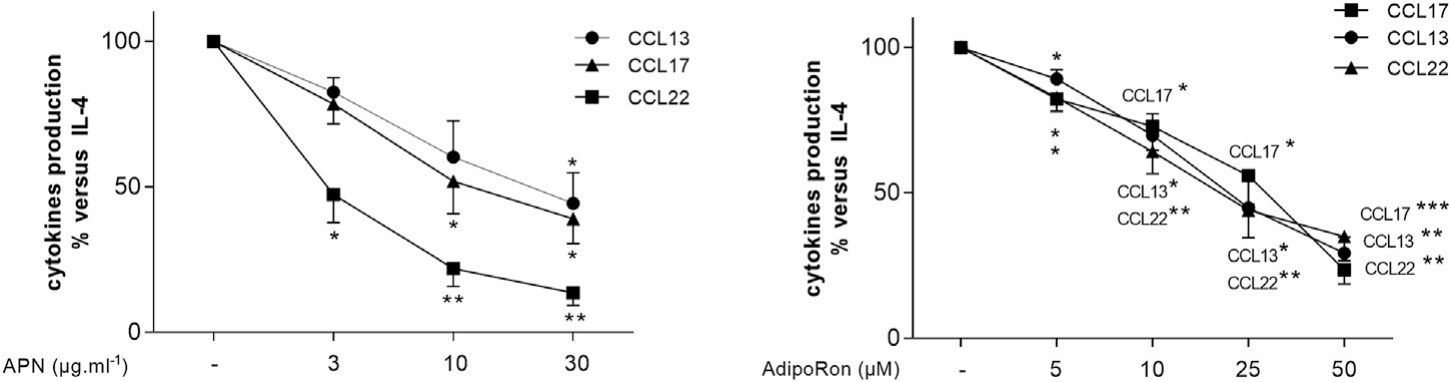
Inhibitory effects of APN and AdipoRon on the IL-4-induced chemokine release by human LMs. Macrophages were incubated in the absence or presence of APN (3, 10 or 30 µg ml^−1^) or AdipoRon (5, 10, 25 or 50 µM) before being stimulated with IL-4 (10 ng ml^−1^) for 24 h. Data are expressed as a percentage with respect to IL-4-induced production. Results are shown as the mean ± SEM of 6–12 different experiments. Asterisks indicate significant effects with respect to IL-4 alone (*:*p* < 0.05; **: *p* < 0.01; ***: *p* < 0.001).

Incubation with IL-4 was associated with the elevated production of the M2-chemokines CCL13 (basal: 16 ± 10 pg ml^−1^; +IL-4: 83 ± 14 pg ml^−1^; a 5.2-fold increase), CCL17 (basal: 6.2 ± 2.1 pg ml^−1^; +IL-4: 46.6 ± 28.7 pg ml^−1^; a 7.5-fold increase) and CCL22 (basal: 0.9 ± 0.7 ng ml^−1^; +IL-4: 3.6 ± 1.6 ng ml^−1^; a 4-fold increase) on 5-6 paired preparations. Both APN and AdipoRon inhibited IL-4-stimulated chemokine production in a concentration-dependent manner (Figure 7 and Supplementary Table S5).

## Discussion

The present study is the first to have demonstrated 1) the production of APN by human lung explants and its correlation with the donor’s BMI, 2) that both AdipoR1 and AdipoR2 are expressed by human LMs, and 3) both APN and the small-molecule agonist AdipoRon reduce the LPS-/poly (I:C)- and the IL-4-induced production of cytokines by LMs.

In patients with asthma, sputum levels of APN were found to be elevated after an allergen challenge (Biagioni et al., 2014). Adiponectin has also been detected in bronchial alveolar lavage (BAL) both in asthma and COPD and was even one of the most strongly expressed cytokines in BAL (Miller et al., 2009; Holguin et al., 2011; Sideleva et al., 2012; Kramer et al., 2017). However, the APN concentrations in BAL were several magnitudes lower than those found in the serum, which probably reflects the dilution of lung fluids by the BAL process. It has been suggested that most of the adipokines in BAL diffuse from the bloodstream, although other researchers have reported disparities between serum and BAL concentrations of APN (Holguin et al., 2011; Kramer et al., 2017). This disparity might be due to the active transport of circulating APN multimers (which might not pass freely through the pulmonary vasculature into the alveolar space) (Sood 2010) or by local synthesis of APN (independently of the blood concentration). In the present study, we showed that APN is produced by human lung explants but not by latter’s constituent LMs. In the lungs, APN may be also synthesized by the epithelial cells, as suggested by the high levels of APN expressed by the human A549 epithelial cell line (Miller et al., 2009), although the involvement of other cell types cannot be ruled out. Adiponectin is not the only adipokine synthetized by the lung, since leptin is reportedly synthetized by bronchial epithelial cells and alveolar type II pneumocytes (Bruno et al., 2009; Vernooy et al., 2009).

It is widely accepted that serum APN levels are positively associated with age and female gender, and negatively associated with BMI (Arita et al., 1999; Lindberg et al., 2013; Lindberg et al., 2017). However, BAL levels of APN were seen to be weakly and negatively associated with BMI in a small sample of patients with asthma and healthy controls (Holguin et al., 2011). This weak association may reflect variations in the diluting effect of the BAL. In contrast, we found a clear association between APN concentrations in the supernatants of explants and the donor’s BMI – suggesting that lung APN production is related to BMI. After taking account of dilution in the culture medium, the estimated APN concentration in lung tissue is close to the range found in the blood (5–20 µg ml^−1^) and to the concentrations of recombinant APN found to inhibit cytokine production by LMs in the present study.

The expression of the APN receptors AdipoR1 and AdipoR2 has been previously reported on murine macrophage-like cells (RAW264) (Yamaguchi et al., 2005), human monocytes (Kollias et al., 2011), MDMs (Cheng et al., 2012), and the THP-1 cell line (van Stijn et al., 2015). In the present study, we showed for the first time that human LMs express both APN receptors. Unstimulated LMs expressed AdipoR1 more strongly than AdipoR2 – as also reported in human monocytes (Kollias et al., 2011; van Stijn et al., 2015). The activation of mouse bone marrow or peritoneal macrophages by interferon-γ/LPS was associated with downregulation of AdipoR1/R2 transcription (van Stijn et al., 2015); this contrasted with the upregulation observed in human LMs activated by LPS. Hence, APN receptor expression appears to be regulated differently in LPS-activated mouse macrophages vs human LMs. Activation of murine macrophages by IL-4 or IL-10 was associated with 1) weak downregulation of or no change in AdipoR1 expression, and 2) upregulation of or no change in AdipoR2 expression (van Stijn et al., 2015). In human LMs activated by IL-4, AdipoR1 mRNA levels did not vary and AdipoR2 mRNA levels rose.

Previous research has shown that incubation of nonstimulated primary human monocytes or MDMs with recombinant APN produced in mammalian cells is associated with elevated mRNA and/or protein levels of M1 and M2 cytokines such as TNF-α and IL-6, and the chemokines CCL2, -3, -4, and -5, and CCL23 (Neumeier et al., 2011; Cheng et al., 2012). The C-terminal globular fragment of APN has been shown to activate nuclear factor-κB and to increase the production of TNF-α, IL-6 and/or CXCL8 in the U937 monocytic cell line and THP-1 macrophages (Tsatsanis et al., 2005; Haugen et al., 2007). Furthermore, adiponectin’s HMW isoforms have been shown to induce the production of IL-6, CXCL-8 and CCL2 in THP-1 cells and human primary monocytes (Neumeier et al., 2006; Haugen et al., 2007; Song and Chan 2009). In sharp contrast, incubation of nonstimulated LMs either with APN produced in HEK293 cells (containing HMW isoforms) or with recombinant APN from *E. coli* did not increase the production of TNF-α, CCL2, CCL3, CCL4, or CXCL8 (present study). Hence, the differences in composition (LMW *vs* HMW isoforms) between recombinant APNs are unlikely to explain the different effects on nonstimulated monocytic cells and LMs. Moreover, the synthetic APN receptor agonist AdipoRon did not induce the production of cytokines by nonstimulated LMs. Therefore, the absence of APN-associated pro-inflammatory effect on LMs is probably due to differences in the cell types rather than in the effects of LMW vs HMW isoforms. Indeed, monocytes and MDM are surrogate cell models that do not adequately recapitulate the biology of LM - as demonstrated in our laboratory (Victoni et al., 2017).

We also showed for the first time that APN from *E. coli* exerts an anti-inflammatory effect on human LMs by largely decreasing the production of cytokines induced by LPS, poly (I:C) or IL-4 without significant impact on cell viability. Such a reduction level in the cytokine release by human monocytes without any impact on cell viability has already been demonstrated with prostaglandin E_2_ (Takayama et al., 2002; Bryn et al., 2006). In addition, we have recently shown a similar magnitude of the APN-induced reduction in cytokine production by human primary bronchial epithelial cells (Salvator et al., 2020). In previous research, eukaryotic recombinant APN was found to have an anti-inflammatory effect 1) in the human THP-1 monocytic cell line (by repressing several TNF-α-induced proinflammatory genes, including cytokines such as CXCL8 (van Stijn et al., 2015)), 2) in the U-937 monocytic cell line (by inhibiting LPS-induced NF-κB activity (Haugen et al., 2007)), 3) in human monocytes or MDMs (by either suppressing the expression or production of LPS-induced cytokines such as TNF-α, IL-6, CXCL9-11 (Neumeier et al., 2006; Okamoto et al., 2008; Folco et al., 2009)). Although APN expressed in HEK293 cells inhibited TNF-α and IL-6 production in MDMs (Folco et al., 2009), it is noteworthy that this type of eukaryotic recombinant APN weakly suppressed LPS-induced cytokine production by LMs in the present study. It is worth noting that HMW-APN did not suppress LPS-induced IL-6 production in human monocytes and THP-1 cells while LMW-APN reduced LPS-mediated IL-6 release (Neumeier et al., 2006). Furthermore, mouse recombinant APN produced in *E. coli* inhibited the LPS-mediated release of TNF-α by mouse alveolar macrophages (Summer et al., 2008). The results of our experiments on batches of APN from *E. coli* that differed with respect to the LMW isoform profile suggested that the latter parameter is involved in APN’s anti-inflammatory effect in LMs. In addition, the observed inhibitory effect of AdipoRon on LPS-, poly (I:C) and IL-4-induced cytokine production confirmed that APN receptor activation has anti-inflammatory effects on LMs.

Adiponectin has been shown to stimulate IL-10 expression and production by human blood monocytes and MDMs (Neumeier et al., 2006; Folco et al., 2009). However, antagonism of IL-10 did not abrogate APN’s anti-inflammatory actions - indicating that the inhibition of cytokine production in response to various proinflammatory stimuli does not require IL-10 (Folco et al., 2009). In contrast to APN’s stimulatory effect on MDMs, APN decreased the LPS-induced production of IL-10 by LMs in the present study. The reasons for this discrepancy remain unclear, although the simplest explanation is that we used human LMs and the previous studies used human monocytes or MDMs (Neumeier et al., 2006; Folco et al., 2009).

Our present findings indicate that LMW APN may act to maintain LMs in a quiescent state and thus protect the lung from dysregulated macrophage activation. These results and our previous results in human bronchial epithelial cells (Salvator et al., 2020) also suggest that the reduction in APN levels associated with an elevated BMI could be a risk factor for the development of inflammatory lung diseases. Asthma associated with obesity is a growing public health problem for which there are few effective treatments (Umetsu 2017) and recent COVID-19 pandemic highlighted once again susceptibility of obese persons towards serious viral infections. The activation of APN receptors with inhaled small molecule agonists may constitute a new pharmacological means of controlling the airways inflammation both in chronic pulmonary inflammatory diseases and acute pulmonary viral infections.

## Data Availability

The raw data supporting the conclusions of this article will be made available by the authors, without undue reservation.

## Abbreviations

AdipoR, adiponectin receptor; APN, adiponectin; BMI, body mass index; FEV1, forced expiratory volume in one second; FVC, forced vital capacity; IL, Interleukin; LM, lung macrophage; MDM, monocyte-derived macrophage; TLR, toll-like receptor.

## References

[B1] Abrial C. Grassin-Delyle S. Salvator H. Brollo M. Naline E. Devillier P. (2015). 15-Lipoxygenases Regulate the Production of Chemokines in Human Lung Macrophages. Br. J. Pharmacol. 172 (17), 4319–4330. 10.1111/bph.13210 26040494 PMC4556470

[B2] GBD 2015 Obesity Collaborators Afshin A. Forouzanfar M. H. Reitsma M. B. Sur P. Estep K. Lee A. (2017). Health Effects of Overweight and Obesity in 195 Countries over 25 Years. N. Engl. J. Med. 377 (1), 13–27. 10.1056/NEJMoa1614362 28604169 PMC5477817

[B3] Arita Y. Kihara S. Ouchi N. Takahashi M. Maeda K. Miyagawa J. (1999). Paradoxical Decrease of an Adipose-specific Protein, Adiponectin, in Obesity. Biochem. Biophys. Res. Commun. 257 (1), 79–83. 10.1006/bbrc.1999.0255 10092513

[B4] Baik I. Curhan G. C. Rimm E. B. Bendich A. Willett W. C. Fawzi W. W. (2000). A Prospective Study of Age and Lifestyle Factors in Relation to Community-Acquired Pneumonia in US Men and Women. Arch. Intern. Med. 160 (20), 3082–3088. 10.1001/archinte.160.20.3082 11074737

[B5] Biagioni B. J. Pui M. M. Fung E. Wong S. Hosseini A. Dybuncio A. (2014). Sputum Adiponectin as a Marker for Western Red Cedar Asthma. J. Allergy Clin. Immunol. 134 (6), 1446–e5. 10.1016/j.jaci.2014.06.037 25129682

[B6] Bruno A. Pace E. Chanez P. Gras D. Vachier I. Chiappara G. (2009). Leptin and Leptin Receptor Expression in Asthma. J. Allergy Clin. Immunol. 124 (2), 230–234. 10.1016/j.jaci.2009.04.032 19539983

[B7] Bryn T. Mahic M. Enserink J. M. Schwede F. Aandahl E. M. Taskén K. (2006). The Cyclic AMP-Epac1-Rap1 Pathway Is Dissociated from Regulation of Effector Functions in Monocytes but Acquires Immunoregulatory Function in Mature Macrophages. J. Immunol. 176 (12), 7361–7370. 10.4049/jimmunol.176.12.7361 16751380

[B8] Buenestado A. Chaumais M. C. Grassin-Delyle S. Risse P. A. Naline E. Longchampt E. (2013). Roflumilast Inhibits Lipopolysaccharide-Induced Tumor Necrosis Factor-α and Chemokine Production by Human Lung Parenchyma. PLoS One 8 (9), e74640. 10.1371/journal.pone.0074640 24066150 PMC3774805

[B9] Buenestado A. Grassin Delyle S. Arnould I. Besnard F. Naline E. Blouquit-Laye S. (2010). The Role of Adenosine Receptors in Regulating Production of Tumour Necrosis Factor-Alpha and Chemokines by Human Lung Macrophages. Br. J. Pharmacol. 159 (6), 1304–1311. 10.1111/j.1476-5381.2009.00614.x 20136829 PMC2848934

[B10] Buenestado A. Grassin-Delyle S. Guitard F. Naline E. Faisy C. Israël-Biet D. (2012). Roflumilast Inhibits the Release of Chemokines and TNF-α from Human Lung Macrophages Stimulated with Lipopolysaccharide. Br. J. Pharmacol. 165 (6), 1877–1890. 10.1111/j.1476-5381.2011.01667.x 21913898 PMC3372837

[B11] Cheng X. Folco E. J. Shimizu K. Libby P. (2012). Adiponectin Induces Pro-inflammatory Programs in Human Macrophages and CD4+ T Cells. J. Biol. Chem. 287 (44), 36896–36904. 10.1074/jbc.M112.409516 22948153 PMC3481292

[B12] Folco E. J. Rocha V. Z. López-Ilasaca M. Libby P. (2009). Adiponectin Inhibits Pro-inflammatory Signaling in Human Macrophages Independent of Interleukin-10. J. Biol. Chem. 284 (38), 25569–25575. 10.1074/jbc.M109.019786 19617629 PMC2757958

[B13] Haugen F. Drevon C. A. (2007). Activation of Nuclear Factor-KappaB by High Molecular Weight and Globular Adiponectin. Endocrinology 148 (11), 5478–5486. 10.1210/en.2007-0370 17702846

[B14] Holguin F. Rojas M. Brown L. A. Fitzpatrick A. M. (2011). Airway and Plasma Leptin and Adiponectin in Lean and Obese Asthmatics and Controls. J. Asthma 48 (3), 217–223. 10.3109/02770903.2011.555033 21332421 PMC3085138

[B15] Kass D. A. Duggal P. Cingolani O. (2020). Obesity Could Shift Severe COVID-19 Disease to Younger Ages. Lancet 395 (10236), 1544–1545. 10.1016/S0140-6736(20)31024-2 32380044 PMC7196905

[B16] Kollias A. Tsiotra P. C. Ikonomidis I. Maratou E. Mitrou P. Kyriazi E. (2011). Adiponectin Levels and Expression of Adiponectin Receptors in Isolated Monocytes from Overweight Patients with Coronary Artery Disease. Cardiovasc. Diabetol. 10 (février), 14. 10.1186/1475-2840-10-14 21284833 PMC3042923

[B17] Kramer M. M. Hirota J. A. Sood A. Teschke K. Carlsten C. (2017). Airway and Serum Adipokines after Allergen and Diesel Exposure in a Controlled Human Crossover Study of Atopic Adults. Transl Res. 182, 49–60. 10.1016/j.trsl.2016.11.001 27886976

[B18] Leal Vde. O. Mafra D. (2013). Adipokines in Obesity. Clin. Chim. Acta 419 (avril), 87–94. 10.1016/j.cca.2013.02.003 23422739

[B19] Lighter J. Phillips M. Hochman S. Sterling S. Johnson D. Francois F. (2020). Obesity in Patients Younger Than 60 Years Is a Risk Factor for Covid-19 Hospital Admission. Clin. Infect. Dis. 71, 896–897. 10.1093/cid/ciaa415 32271368 PMC7184372

[B20] Lindberg S. Jensen J. S. Bjerre M. Frystyk J. Flyvbjerg A. Jeppesen J. (2017). Low Adiponectin Levels at Baseline and Decreasing Adiponectin Levels over 10 Years of Follow-Up Predict Risk of the Metabolic Syndrome. Diabetes Metab. 43 (2), 134–139. 10.1016/j.diabet.2016.07.027 27639310

[B21] Lindberg S. Mogelvang R. Pedersen S. H. Bjerre M. Frystyk J. Flyvbjerg A. (2013). Relation of Serum Adiponectin Levels to Number of Traditional Atherosclerotic Risk Factors and All-Cause Mortality and Major Adverse Cardiovascular Events (From the Copenhagen City Heart Study). Am. J. Cardiol. 111 (8), 1139–1145. 10.1016/j.amjcard.2012.12.043 23375598

[B22] Miller M. Cho J. Y. Pham A. Ramsdell J. Broide D. H. (2009). Adiponectin and Functional Adiponectin Receptor 1 Are Expressed by Airway Epithelial Cells in Chronic Obstructive Pulmonary Disease. J. Immunol. 182 (1), 684–691. 10.4049/jimmunol.182.1.684 19109202

[B23] Morgan O. W. Bramley A. Fowlkes A. Freedman D. S. Taylor T. H. Gargiullo P. (2010). Morbid Obesity as a Risk Factor for Hospitalization and Death Due to 2009 Pandemic Influenza A(H1N1) Disease. PLoS One 5 (3), e9694. 10.1371/journal.pone.0009694 20300571 PMC2837749

[B24] Murray P. J. Allen J. E. Biswas S. K. Fisher E. A. Gilroy D. W. Goerdt S. (2014). Macrophage Activation and Polarization: Nomenclature and Experimental Guidelines. Immunity 41 (1), 14–20. 10.1016/j.immuni.2014.06.008 25035950 PMC4123412

[B25] NCD Risk Factor Collaboration (NCD-RisC) (2017). Worldwide Trends in Body-Mass index, Underweight, Overweight, and Obesity from 1975 to 2016: a Pooled Analysis of 2416 Population-Based Measurement Studies in 128·9 Million Children, Adolescents, and Adults. Lancet 390 (10113), 2627–2642. 10.1016/S0140-6736(17)32129-3 29029897 PMC5735219

[B26] Neumeier M. Bauer S. Brühl H. Eisinger K. Kopp A. Abke S. (2011). Adiponectin Stimulates Release of CCL2, -3, -4 and -5 while the Surface Abundance of CCR2 and -5 Is Simultaneously Reduced in Primary Human Monocytes. Cytokine 56 (3), 573–580. 10.1016/j.cyto.2011.08.017 21890375

[B27] Neumeier M. Weigert J. Schäffler A. Wehrwein G. Müller-Ladner U. Schölmerich J. (2006). Different Effects of Adiponectin Isoforms in Human Monocytic Cells. J. Leukoc. Biol. 79 (4), 803–808. 10.1189/jlb.0905521 16434692

[B28] Ohashi K. Parker J. L. Ouchi N. Higuchi A. Vita J. A. Gokce N. (2010). Adiponectin Promotes Macrophage Polarization toward an Anti-inflammatory Phenotype. J. Biol. Chem. 285 (9), 6153–6160. 10.1074/jbc.M109.088708 20028977 PMC2825410

[B29] Ohashi K. Shibata R. Murohara T. Ouchi N. (2014). Role of Anti-inflammatory Adipokines in Obesity-Related Diseases. Trends Endocrinol. Metab. 25 (7), 348–355. 10.1016/j.tem.2014.03.009 24746980

[B30] Okada-Iwabu M. Yamauchi T. Iwabu M. Honma T. Hamagami K. Matsuda K. (2013). A Small-Molecule AdipoR Agonist for Type 2 Diabetes and Short Life in Obesity. Nature 503 (7477), 493–499. 10.1038/nature12656 24172895

[B31] Okamoto Y. Folco E. J. Minami M. Wara A. K. Feinberg M. W. Sukhova G. K. (2008). Adiponectin Inhibits the Production of CXC Receptor 3 Chemokine Ligands in Macrophages and Reduces T-Lymphocyte Recruitment in Atherogenesis. Circ. Res. 102 (2), 218–225. 10.1161/CIRCRESAHA.107.164988 17991878

[B32] Ouchi N. Parker J. L. Lugus J. J. Walsh K. (2011). Adipokines in Inflammation and Metabolic Disease. Nat. Rev. Immunol. 11 (2), 85–97. 10.1038/nri2921 21252989 PMC3518031

[B33] Pechkovsky D. V. Prasse A. Kollert F. Engel K. M. Dentler J. Luttmann W. (2010). Alternatively Activated Alveolar Macrophages in Pulmonary Fibrosis-Mediator Production and Intracellular Signal Transduction. Clin. Immunol. 137 (1), 89–101. 10.1016/j.clim.2010.06.017 20674506

[B34] Salvator H. Devillier P. Rivaud E. Catherinot E. Honderlick P. Couderc L. J. (2011). Obesity, Poor Prognostic Factor in Pandemic Influenza A (H1N1) 2009: the Role of Adipokines in the Modulation of Respiratory Defenses. Rev. Pneumol Clin. 67 (4), 244–249. 10.1016/j.pneumo.2011.01.001 21920285

[B35] Salvator H. Grassin-Delyle S. Naline E. Brollo M. Fournier C. Couderc L. J. (2020). Contrasting Effects of Adipokines on the Cytokine Production by Primary Human Bronchial Epithelial Cells: Inhibitory Effects of Adiponectin. Front. Pharmacol. 11, 56. 10.3389/fphar.2020.00056 32132922 PMC7040162

[B36] Shapouri-Moghaddam A. Mohammadian S. Vazini H. Taghadosi M. Esmaeili S. A. Mardani F. (2018). Macrophage Plasticity, Polarization, and Function in Health and Disease. J. Cel. Physiol. 233 (9), 6425–6440. 10.1002/jcp.26429 PMC13482560 29319160

[B37] Shaykhiev R. Krause A. Salit J. Strulovici-Barel Y. Harvey B. G. O'Connor T. P. (2009). Smoking-Dependent Reprogramming of Alveolar Macrophage Polarization: Implication for Pathogenesis of Chronic Obstructive Pulmonary Disease. J. Immunol. 183 (4), 2867–2883. 10.4049/jimmunol.0900473 19635926 PMC2873685

[B38] Sideleva O. Suratt B. T. Black K. E. Tharp W. G. Pratley R. E. Forgione P. (2012). Obesity and Asthma: An Inflammatory Disease of Adipose Tissue Not the Airway. Am. J. Respir. Crit. Care Med. 186 (7), 598–605. 10.1164/rccm.201203-0573OC 22837379 PMC3480522

[B39] Song H. Chan J. Rovin B. H. (2009). Induction of Chemokine Expression by Adiponectin *In Vitro* Is Isoform Dependent. Transl Res. 154 (1), 18–26. 10.1016/j.trsl.2009.04.003 19524870 PMC2727280

[B40] Sood A. (2010). Obesity, Adipokines, and Lung Disease. J. Appl. Physiol. (1985) 108 (3), 744–753. 10.1152/japplphysiol.00838.2009 19926824 PMC2838636

[B41] Staples K. J. Hinks T. S. Ward J. A. Gunn V. Smith C. Djukanović R. (2012). Phenotypic Characterization of Lung Macrophages in Asthmatic Patients: Overexpression of CCL17. J. Allergy Clin. Immunol. 130 (6), 1404–e7. 10.1016/j.jaci.2012.07.023 22981793 PMC3805016

[B42] Summer R. Little F. F. Ouchi N. Takemura Y. Aprahamian T. Dwyer D. (2008). Alveolar Macrophage Activation and an Emphysema-like Phenotype in Adiponectin-Deficient Mice. Am. J. Physiol. Lung. Cel. Mol. Physiol. 294 (6), L1035–L1042. 10.1152/ajplung.00397.2007 PMC3575679 18326826

[B43] Sutherland E. R. (2014). Linking Obesity and Asthma. Ann. N. Y Acad. Sci. 1311 (avril), 31–41. 10.1111/nyas.12357 24517401

[B44] Takayama K. García-Cardena G. Sukhova G. K. Comander J. Gimbrone M. A. Libby P. (2002). Prostaglandin E2 Suppresses Chemokine Production in Human Macrophages through the EP4 Receptor. J. Biol. Chem. 277 (46), 44147–44154. 10.1074/jbc.M204810200 12215436

[B45] Tsao T. S. Murrey H. E. Hug C. Lee D. H. Lodish H. F. (2002). Oligomerization State-dependent Activation of NF-Kappa B Signaling Pathway by Adipocyte Complement-Related Protein of 30 KDa (Acrp30). J. Biol. Chem. 277 (33), 29359–29362. 10.1074/jbc.C200312200 12087086

[B46] Tsatsanis C. Zacharioudaki V. Androulidaki A. Dermitzaki E. Charalampopoulos I. Minas V. (2005). Adiponectin Induces TNF-Alpha and IL-6 in Macrophages and Promotes Tolerance to Itself and Other Pro-inflammatory Stimuli. Biochem. Biophys. Res. Commun. 335 (4), 1254–1263. 10.1016/j.bbrc.2005.07.197 16115611

[B47] Umetsu D. T. (2017). Mechanisms by Which Obesity Impacts upon Asthma. Thorax 72 (2), 174–177. 10.1136/thoraxjnl-2016-209130 27672120

[B48] Van Kerkhove M. D. Vandemaele K. A. Shinde V. Jaramillo-Gutierrez G. Koukounari A. Donnelly C. A. (2011). Risk Factors for Severe Outcomes Following 2009 Influenza A (H1N1) Infection: A Global Pooled Analysis. Plos Med. 8 (7), e1001053. 10.1371/journal.pmed.1001053 21750667 PMC3130021

[B49] van Stijn C. M. Kim J. Lusis A. J. Barish G. D. Tangirala R. K. (2015). Macrophage Polarization Phenotype Regulates Adiponectin Receptor Expression and Adiponectin Anti-inflammatory Response. FASEB J. 29 (2), 636–649. 10.1096/fj.14-253831 25392268 PMC4314235

[B50] Vernooy J. H. Drummen N. E. van Suylen R. J. Cloots R. H. Möller G. M. Bracke K. R. (2009). Enhanced Pulmonary Leptin Expression in Patients with Severe COPD and Asymptomatic Smokers. Thorax 64 (1), 26–32. 10.1136/thx.2007.085423 18835960

[B51] Victoni T. Salvator H. Abrial C. Brollo M. Porto L. C. S. Lagente V. (2017). Human Lung and Monocyte-Derived Macrophages Differ with Regard to the Effects of β2-adrenoceptor Agonists on Cytokine Release. Respir. Res. 18 (1), 126. 10.1186/s12931-017-0613-y 28637505 PMC5480184

[B52] Wulster-Radcliffe M. C. Ajuwon K. M. Wang J. Christian J. A. Spurlock M. E. Spurlock M. E. (2004). Adiponectin Differentially Regulates Cytokines in Porcine Macrophages. Biochem. Biophys. Res. Commun. 316 (3), 924–929. 10.1016/j.bbrc.2004.02.130 15033490

[B53] Yamaguchi N. Argueta J. G. Masuhiro Y. Kagishita M. Nonaka K. Saito T. (2005). Adiponectin Inhibits Toll-like Receptor Family-Induced Signaling. FEBS Lett. 579 (30), 6821–6826. 10.1016/j.febslet.2005.11.019 16325814

[B54] Yokota T. Oritani K. Takahashi I. Ishikawa J. Matsuyama A. Ouchi N. (2000). Adiponectin, a New Member of the Family of Soluble Defense Collagens, Negatively Regulates the Growth of Myelomonocytic Progenitors and the Functions of Macrophages. Blood 96 (5), 1723–1732. 10.1182/blood.v96.5.1723.h8001723_1723_1732 10961870

