# Supplementary material for: Adiponectin Inhibits the Production of TNF-α, IL-6 and Chemokines by Human Lung Macrophages
Source: Front Pharmacol. 2021 Aug 23;12:718929. doi: 10.3389/fphar.2021.718929 (PMC8428996; doi:10.3389/fphar.2021.718929)
Supplement: Supplementary file 1 [file DataSheet1.doc]

**Supplementary results**

Cytotoxicity assays

Cell viability was assessed by measuring LDH release with the CytoTox96® Non-Radioactive Cytotoxicity Assay (Promega®, Madison, USA). The incubation with adiponectin (3-10-30µg.ml^-1^) or Adiporon (5-10-25-50 µM) was not associated with a significant increase in LDH release by the LMs (n=5).

Inhibitory effect of APN on LPS-induced production of IL-10

Lipopolysaccharide treatment was associated with elevated IL-10 production (mean ± SEM: 1245 ± 328 pg.mL^-1^). The IL-10 production was reduced by APN at 10 µg.mL^-1^ (682 ± 320 pg.ml^-1^, -52%; p<0.05) and 30 µg.mL^-1^ (502 ± 349 .ml^-1^, -70%; p< 0.01).

**Table S1. Amounts of cytokines in the supernatants of unstimulated human primary pulmonary macrophages treated with APN (A), APN produced in HEK293 (B) or Adiporon (C).**

Lung macrophages were incubated with recombinant APN form E. coli (APN, 3 and 10 µg.mL^-1^), APN from HEK293 (APNHK, 3, 10 and 30 µg.mL^-1^) or adipoRon (Aron) (5, 10, 25, 50 µM). Cell culture supernatants were collected after 24h incubation and analyzed by ELISA. Results are expressed in pg.10^-6^cells and are shown as the means ± SEM of 5 independent experiments. * p<0.05 versus “vehicle”.

A.

|  | **vehicle** | **APN 3 µg.mL-1** | **APN 10 µg.mL-1** |
| --- | --- | --- | --- |
| **TNF-α** | **99±41** | **76±30** | **53±18** |
| **CXCL8** | **16348±4269** | **15108±4583** | **14519±3477** |
| **CCL2** | **8005±2848** | **7737±2673** | **7592±3198** |
| **CCL3** | **1052±501** | **1118±565** | **961±488** |
| **CCL4** | **4066±2127** | **3267±1698** | **2833±1375** |

B.

|  | **vehicle** | **APNHK 3 µg.mL-1** | **APNHK 10 µg.mL-1** | **APNHK 30 µg.mL-1** |
| --- | --- | --- | --- | --- |
| **TNF-α** | **42±8** | **37±9** | **41±9** | **39±8** |
| **CXCL8** | **34052±7989** | **33308±7408** | **34276±7622** | **32138±7149** |
| **CCL3** | **2277±697** | **2144±696** | **1986±647** | **1801±545** |
| **CCL4** | **6432±2305** | **6376±2320** | **6137±2122** | **6490±2423** |

C.

|  | **vehicle** | **Aron 5 µM** | **Aron 10 µM** | **Aron 25 µM** | **Aron 50 µM** |
| --- | --- | --- | --- | --- | --- |
| **TNF-α** | **85±23** | **78±22** | **70±17** | **51±14*** | **25±6*** |
| **CXCL8** | **40236±3953** | **38786±4775** | **37392±3709** | **36272±3439** | **29525±3157** |
| **CXCL1** | **2354±709** | **2177±635** | **1955±576** | **1691±646** | **1296±413** |
| **IL-6** | **950±420** | **790±370** | **593±291*** | **560±309*** | **294±175*** |

**Table S2. Amounts of cytokines in the supernatants of human primary LMs treated with LPS (A) or Poly I:C (B) and adiponectin.**

Lung macrophages were incubated with (A) LPS alone (10 ng.mL^-1^) or adiponectin (APN) (3, 10, 30 µg.mL^-1^) + LPS (10 ng.mL^-^1, added one hour after APN) and with (B) poly(I:C) or adiponectin (APN) (3, 10, 30 µg.mL^-1^) + poly(I:C) (10 µg.mL^-1^, added one hour after APN). Cell culture supernatants were collected after 24h incubation and analyzed by ELISA. Results are expressed in ng.10^6^cells and are shown as the means ± SEM of 5-16 independent experiments. * p<0.05; ** p< 0.01; *** p<0.001, *versus* “LPS alone” or “Poly(I:C) alone”.

**A.**

|  | **LPS alone** | **LPS + APN 3µg.mL-1** | | **LPS + APN 10µg.mL-1** | | **LPS + APN 30µg.mL-1** | |
| --- | --- | --- | --- | --- | --- | --- | --- |
|  |  |  | **% inhibition vs LPS alone** |  | **% inhibition vs LPS alone** |  | **% inhibition vs LPS alone** |
| **CCL3 n=12** | 171.6±33.6 | 146.9±32.5  * | 16% | 87.1±26.6  ** | 52% | 1.2±0.3  *** | -99% |
| **CCL4 n=12** | 270.1±47.9 | 215.2±34.0  * | 17% | 105.3±24.1  ** | 56% | 1.3±0.7  *** | 99% |
| **CCL5 n=14** | 2.6±0.7 | 2.0±0.6  ** | 23% | 1.3±0.4  *** | 55% | 0.1±0.1  *** | 96% |
| **CXCL1 n=9** | 178.4±32.2 | 139.9±23.5 | 18% | 52.5±13.5  ** | 68% | 10.6±4.1  ** | 92% |
| **CXCL8 n=14** | 940.6±201.8 | 793.2±214  * | 23% | 544.9±172.3  *** | 52.7% | 9.3±4.3  *** | 99% |
| **CXCL10 n=5** | 1.3±0.7 | 0.6±0.3  * | 48% | 0.4±0.2  * | 64% | 0.2±0.1  ** | 85% |
| **TNF-α**  **n=16** | 17.8±3.4 | 16.0±3.7 | 16% | 12.0±3.2  * | 34% | 1.2±0.7  *** | 93% |
| **IL-6 n=9** | 76±23.6 | 65.2±20.8 | 17% | 42.0±16.6  ** | 57% | 0.3±0.2  *** | 97% |

**B.**

|  | **POLY(I:C) alone** | **POLY(I:C) + APN 3µg.mL^-1^** | | **POLY(I:C) + APN 10µg.mL^-1^** | | **POLY(I:C) + APN 30µg.mL^-1^** | |
| --- | --- | --- | --- | --- | --- | --- | --- |
|  |  |  | **% inhibition vs POLY(I:C) alone** |  | **% inhibition vs POLY(I:C) alone** |  | **% inhibition vs POLY(I:C) alone** |
| **CCL3 n=5** | 52.7±16.2 | 38.2±12.2 | 24% | 13.3±8.0  * | 72% | 0.87±0.51  *** | 99% |
| **CCL4 n=5** | 224.6±36 | 131.8±17  * | 38% | 43.6±17.6  * | 76% | 0.91±0.7  ** | 99% |
| **CCL5 n=5** | 4.7±1.2 | 2.8±0.4 | 33% | 1±0.3  * | 73% | 0.05±0.04  ** | 99% |
| **CXCL1 n=5** | 5.3±1.1 | 5±1.2 | 7% | 2.8±0.8  * | 48% | 0.34±0.2  ** | 94% |
| **CXCL8 n=5** | 84.8±16.9 | 83.5±16.1 | 0% | 71.6±12.4 | 13% | 8.5±1.9  *** | 90% |
| **CXCL10 n=5** | 14.6±2.3 | 12.5±2.3 | 15% | 2.9±1.5  * | 80% | 0.1±0.04  ** | 99% |
| **TNFα n=5** | 8.5±1.8 | 5.2±1.6 | 26% | 3.7±1.4 | 48% | 0.52±0.2  ** | 92% |
| **IL6 n=5** | 5.0±1.3 | 3.9±1.4 | 20% | 2.2±0.9 | 49% | 0.22±0.12  ** | 96% |

**Table S3. Amounts of cytokines in the supernatants of human primary pulmonary macrophages treated with LPS and APN produced in HEK293.**

Lung macrophages were incubated with LPS alone (10 ng.mL^-1^) or APN from HEK293 (APNHK, 3, 10 and 30 µg.mL^-1^) + LPS (10 ng.mL^-1^, added one hour after APNHEK). Cell culture supernatants were collected after 24h incubation and analyzed by ELISA. Results are expressed in ng.10^-6^ cells and are shown as the means ± SEM of 5 independent experiments. * p<0.05 versus “LPS alone”.

|  | **LPS alone** | **LPS +**  **APNHK 3 µg.mL-1** | **LPS +**  **APNHK 10 µg.mL-1** | **LPS +**  **APNHK 30 µg.mL-1** |
| --- | --- | --- | --- | --- |
| **TNF-α** | 42.1±32.0 | 41.9±31.6 | 38.7±29.7 | 33.1±26.7* |
| **CXCL8** | 2318.6±175.5 | 2162.9±164.8 | 2195.7±169.7 | 1466.7±351.3 |
| **CCL4** | 430.5±145.2 | 406.1±148.2 | 404.5±136.2 | 336.2±128.5* |

**Table S4. Amounts of cytokines in the supernatants of human primary pulmonary macrophages treated with LPS (A) or Poly I:C (B) and AdipoRon.**

Lung macrophages were incubated with (A) LPS alone (10 ng.mL^-1^) or AdipoRon (Aron) (5, 10, 25, 50 µM) + LPS (10 ng.mL^-1^, added one hour after Aron) and with (B) poly(I:C) or AdipoRon (Aron) (5, 10, 25, 50 µM) + LPS (10 ng.mL^-1^, added one hour after Aron). Cell culture supernatants were collected after 24h incubation and analyzed by ELISA. Results are expressed in ng.10^6^ cells and are shown as the means ± SEM of 5-10 independent experiments. * p<0.05; ** p< 0.01; *** p<0.001, *versus* “LPS alone” or “Poly(I:C) alone”.

**A.**

|  | **LPS alone** | **LPS**  **+ Aron 5µM** | **LPS**  **+ Aron 10µM** | | **LPS**  **+ Aron 25µM** | | **LPS**  **+ Aron 50µM** | |
| --- | --- | --- | --- | --- | --- | --- | --- | --- |
|  |  |  |  | **% inhibition vs LPS alone** |  | **% inhibition vs LPS alone** |  | **% inhibition vs LPS alone** |
| **CCL3 n=8** | 224.5±35.9 | 196.4±25.3 | 200.1±29.2 | 15% | 143.2±23.7  * | 41% | 32.1±10.2  *** | 85% |
| **CCL4 n=5** | 222.7±32.2 | 210.9±32.7 | 192.6±24.6 | 13% | 140.6±24 * | 34% | 25.3±8.4  *** | 89% |
| **CCL5 n=7** | 2.3±0.5 | 2.4±0.5 | 2.3±0.5 | 2% | 1.4±0.3  * | 43% | 0.3±0.1  *** | 86% |
| **CXCL1 n=8** | 250.0±60.3 | 220.8±34.6 | 176.5±37.3 | 27% | 110.7±34.4  ** | 63% | 25.3±20.1  ** | 92% |
| **CXCL8 n=8** | 1075.4±242.2 | 1063.4±228.4 | 1084.7±250.8 | 2% | 859.8±249.6 | 27% | 223.5±83.9  ** | 79% |
| **TNF-α n=8** | 50.3±10.3 | 62.5±20.3 | 55.6±12.5 | -12% | 40.1±10.7 | 20% | 4.9±1.1  *** | 88% |
| **IL-6 n=5** | 118.8±19.0 | 107.0±19.8 | 106.7±20.2 | 12% | 59.3±20.9  * | 55% | 5.0±2.1  ** | 96% |

**B.**

|  | **POLY(I:C)**  **alone** | **POLY(I:C)**  **+ Aron 5µM** | **POLY(I:C)**  **+ Aron 10µM** | | **POLY(I:C)**  **+ Aron 25µM** | | **POLY(I:C)**  **+ Aron 50µM** | |
| --- | --- | --- | --- | --- | --- | --- | --- | --- |
|  |  |  |  | **% inhibition vs POLY(I:C) alone** |  | **% inhibition vs POLY(I:C) alone** |  | **% inhibition vs POLY(I:C) alone** |
| **CCL3**  **n=8** | 94.3±14.8 | 91.4±17.1 | 79.1±19.7 | 22% | 44.6±14.3  * | 56% | 14.8±9.2  *** | 86% |
| **CCL4**  **n=8** | 185.2±33.6 | 172.4±37.8 | 134.6±33.1 | 22% | 67.1±22.9  * | 62% | 21.3±12.8  ** | 90% |
| **CCL5**  **n=8** | 4.3±1 | 4.3±1.2 | 3.8±1 | 16% | 1.3±0.4  ** | 67% | 0.3±0.1  ** | 91% |
| **CXCL1**  **n=8** | 11.4±2.1 | 10.9±2.5 | 7.8±2 | 34% | 5.2±1.6  * | 57% | 1.9±0.8  *** | 85% |
| **CXCL8**  **n=5** | 118.8±19 | 107.0±19.8 | 106.7±20.2 | -12% | 59.3±20.9  * | 55% | 5.0±2.0  ** | 96% |
| **TNF-α n=8** | 8.7±1.1 | 9.9±1.6 | 8±1.4 | 9% | 4±1.3  * | 56% | 1.2±0.9  *** | 90% |
| **IL6**  **n=9** | 8.0±1.8 | 7.8±1.9 | 6.0±1.6 | 27% | 2.6±1.3  ** | 70% | 0.5±0.1  *** | 92% |

**Table S5. Amounts of cytokines in the supernatants of primary human pulmonary macrophages treated with IL-4 and adiponectin (A) or adiporon (B).**

Lung macrophages were incubated with (A) IL-4 alone (10 ng.mL^-1^) or in combination with (A) adiponectin (APN) (3, 10, 30 µg.mL^-1^) or (B) adiporon (Aron) (5, 10, 25, 50 µM). IL-4 (10 ng.mL^-1^ was added one hour after APN or Aron). Cell culture supernatants were collected after 24h incubation and analyzed by ELISA. Results are expressed in pg.10^-6^cells and are shown as the means ± SEM of 6-12 independent experiments. * p<0.05; ** p< 0.01; *** p<0.001, *versus* “IL-4 alone”.

A.

|  | **IL-4 alone** | **IL-4 + APN 3µg.mL^-1^** | | **IL-4 + APN 10µg.mL^-1^** | | **IL-4 + APN 30µg.mL^-1^** | |
| --- | --- | --- | --- | --- | --- | --- | --- |
|  |  |  | **% inhibition vs IL-4 alone** |  | **% inhibition vs IL-4 alone** |  | **% inhibition vs IL-4 alone** |
| **CCL13 n=6** | 88.3±12.0 | 71.9±9.5 | 17% | 51.1±13.5 | 40% | 38.2±11.4  * | 56% |
| **CCL17 n=8** | 20.6±7.5 | 17.4±6.6 | 22% | 8.5±2.3  * | 49% | 5.2±1.3  * | 61% |
| **CCL22 n=8** | 3738±1381 | 1690±865  * | 53% | 592±225  ** | 78% | 382±185  ** | 86% |

**B.**

|  | **IL-4** | **IL-4**  **+Aron 5µM** | | **IL-4**  **+ Aron 10µM** | | **IL-4**  **+ Aron 25µM** | | **IL-4**  **+ Aron 50µM** | |
| --- | --- | --- | --- | --- | --- | --- | --- | --- | --- |
|  |  |  | **% inhibition vs IL-4 alone** |  | **% inhibition vs IL-4 alone** |  | **% inhibition vs IL-4 alone** |  | **% inhibition vs IL-4 alone** |
| **CCL13**  **n=9** | 55.3±18 | 48.0±16.2  * | 11% | 38.6±14.8  * | 30% | 21.7±7.9  * | 55% | 16.8±6.4  ** | 71% |
| **CCL17 n=12** | 64.1±27.6 | 51.5±22.4  * | 17% | 53.7±25.1  * | 27% | 41.3±20.3  * | 44% | 19.1±9.9  *** | 76% |
| **CCL22 n=12** | 2583±1158 | 2663±1155  * | 17% | 2304±1042  ** | 36% | 1963±1112  ** | 56% | 1561±888  ** | 65% |

**Figure S1. Composition of batches of adiponectin, considering their content in different isoforms**

4-20% SDS-PAGE separation of human adiponectin provided by the producer (Biovendor):

Column 1 and 4: Molecular weight markers - 14, 21, 31, 45, 66, 97 kDa

Column 2: APN produced in HEK293, reduced and boiled sample, 2.5 μg/lane

Column 3: APN produced in HEK293, non-reduced and non-boiled sample, 2.5 μg/lane

Column 5: APN produced in *E. coli* batch AP-13-028, non-reduced and non-boiled sample,

2.5 μg/lane

Column 6: APN produced in *E. coli* batch AP-16-042, non-reduced and non-boiled sample,

2.5 μg/lane

Comment: The adiponectin produced in HEK293 was inactive or weakly active on LMs.

*E. coli* batch AP-13-028 was active on LM but *E. coli* batch AP-16-042 was inactive on LM
